# The effect of high glucose on the inhibitory action of C21, a selective AT2R agonist, of LPS-stimulated tissue factor expression in human mononuclear cells

**DOI:** 10.1186/s12950-016-0123-6

**Published:** 2016-05-04

**Authors:** Cristina Balia, Valentina Scalise, Silvana Cianchetti, Francesca Faita, Tommaso Neri, Vittoria Carnicelli, Riccardo Zucchi, Alessandro Celi, Roberto Pedrinelli

**Affiliations:** Dipartimento di Patologia Chirurgica, Medica, Molecolare e dell’Area Critica, Università di Pisa, Lungarno Pacinotti 43, 56126 Pisa, Italy

**Keywords:** Tissue factor, Thrombosis, Renin angiotensin system, Innate immunity endotoxin, Inflammation, Renin angiotensin blockers

## Abstract

**Background:**

Intimate links connect tissue factor (TF), the principal initiator of the clotting cascade, to inflammation, a cross-talk amplified by locally generated Angiotensin (AT) II, the effector arm of the Renin Angiotensin System (RAS). C21, a selective AT2R agonist, downregulates the transcriptional expression of TF in LPS-activated peripheral blood mononuclear cell(PBMC)s implying the existence of ATII type 2 receptor (AT2R)s whose stimulation attenuates inflammation-mediated procoagulant responses. High glucose, by activating key signalling pathways and increasing the cellular content of RAS components, augments TF expression and potentiates the inhibitory effect of AT1R antagonists. It is unknown, however, the impact of that stimulus on AT2R-mediated TF inhibition, an information useful to understand more precisely the role of that signal transduction pathway in the inflammation-mediated coagulation process. TF antigen (ELISA), procoagulant activity (PCA, 1-stage clotting assay) and TF-mRNA (real-time polymerase chain reaction) were assessed in PBMCs activated by LPS, a pro-inflammatory and procoagulant stimulus, exposed to either normal (N) or HG concentrations (5.5 and 50 mM respectively).

**Results:**

HG upregulated TF expression, an effect abolished by BAY 11-7082, a NFκB inhibitor. C21 inhibited LPS-stimulated PCA, TFAg and mRNA to an extent independent of glucose concentration but the response to Olmesartan, an AT1R antagonist, was quite evidently potentiated by HG.

**Conclusions:**

HG stimulates LPS-induced TF expression through mechanisms completely dependent upon NFkB activation. Both AT2R-stimulation and AT1R-blockade downregulate inflammation-mediated procoagulant response in PBMCs but HG impacts differently on the two different signal transduction pathways.

## Background

An extensive cross-talk connects coagulation and inflammation [1], a process hinging around Tissue Factor (TF), the principal initiator of the clotting cascade and a major regulator of haemostasis and thrombosis rapidly inducible by inflammatory agents in several cell lines including monocytes [2]. Activation of NFkB, a key redox-sensitive transcription factor encoding for the TF gene [2, 3], plays a key role in that mechanism amplified by locally synthesized Angiotensin (AT) II [4], the final effector of the renin angiotensin system (RAS) and an inflammatory agent on its own [5]. ATII-mediated stimulation of TF expression has consistently been shown in monocytes [6], an immunocompetent cell lineage endowed with the whole biochemical machinery for the endogenous production of angiotensin (AT)II e.g. [7–10] as well as ATII type 1 (AT1R) and type 2 (AT2R) receptors (e.g. [11] eligible for paracrine and/or intracrine stimulation. In agreement with that concept, AT1R antagonists downregulate activated TF expression [12, 13] among others) but the inhibitory effect of selective AT2R stimulation [14] is suggestive of the existence of AT2Rs counteracting the AT1R-mediated procoagulant phenotype, as reported for other AT2R-mediated functions [15].

High glucose (HG) concentrations, by accelerating reactive oxygen species (ROS) generation and increasing NFkB-induced cytokine production e.g. [16–18], increase TF expression in human peripheral blood mononuclear cells (PBMC)s, amplify procoagulant responses in cells activated by endotoxin (Lipopolysaccharide, LPS) and potentiate the inhibitory action of RAS blockers including AT1R antagonists [13]. However, the influence of high glucose environment, an experimental condition reproducing, to some extent, the diabetic state hallmark, on the TF modulation induced by AT2R agonism is unknown, a piece of evidence useful to understand in more detail the role of the AT2R signal transduction pathway in the inflammation-mediated coagulation process.

For this reason, we investigated the effect of C21, a recently synthesized selective AT2R agonist [19], on TF expression in human PBMCs exposed to HG and activated by lipopolysaccharide (LPS, endotoxin), a well characterized target of the innate immune system [20] and a procoagulant agent [2]. Olmesartan (OLM), a selective AT1R antagonist [21], was used as a control.

## Methods

### Cell isolation and culture

Human PBMCs were obtained from unpooled buffy coats left over from blood bank draws taken from healthy donors with the approval of the local ethics committee. According to local procedures, individuals with a history of diabetes and hypertension, either on antihypertensive drugs or not, are excluded from blood donation. As detailed elsewhere [22], leukocytes were isolated from fresh buffy coats diluted 1:1 with sodium citrate 0.38 % in saline solution, mixed gently with 0.5 volume of 2 % Dextran T500 and left for 40 min for erythrocyte sedimentation. The leukocyte-rich supernatant was recovered and centrifuged for 10 min at 200xg. The pellet was resuspended in 30 ml of sodium citrate solution, layered over 15 ml of Ficoll-Hypaque and centrifuged for 30 min at 350xg at 20 °C. The PBMC-rich ring was recovered, washed twice in sodium citrate 0.38 % and resuspended in no glucose RPMI 1640 medium (Sigma Chemical, St Louis, Missouri, USA) supplemented with 100 U/ml penicillin-streptomycin. The final PBMC preparations typically contain 25–35 % monocytes, 65–75 % lymphocytes and less than 5 % neutrophils [22].

After isolation, cells resuspended in polypropylene tubes (3×10^6^ cells/ml) were exposed to experimental drugs or their appropriate vehicle in presence of two different D-glucose concentrations (5.5 mM, hereafter referred to as Normal Glucose, NG, or 50 mM heretofore referred to as High Glucose, HG) 30 min prior to LPS (Escherichia coli 026:B6 LPS; Sigma Chemical), 100 ng/ml, and left to incubate for 18 hs at 37 °C in a 5 % CO_2_ atmosphere until assay. Previous experiments had shown the D-glucose concentration-dependent increase in TF procoagulant activity (see below) as well as the neutral effect of L-Glucose used as a control for osmolarity changes [13] and, therefore, were not repeated. Untreated PBMCs were also included in each experimental series to obtain baseline values. All reagents and solutions used for cell isolation and culture were prepared with endotoxin-free water and glassware was rendered endotoxin-free by exposure to high temperature. Drugs were kept in stock solution and diluted in serum-free RPMI at the appropriate concentrations immediately before use. Cell viability as assessed by dimethyl thiazolyl diphenyl tetrazolium (MTT) was verified (85 % or more of viable cells) throughout all experimental phases.

### Experimental methods

#### TF procoagulant activity (PCA)

PCA was assessed by a one-stage clotting time test in PBMCs disrupted by three freeze–thaw cycles, as previously described [23]. In brief, disrupted cells (100 μl) were mixed with 100 μl of normal human plasma at 37 °C, adding 100 μl of 25 mmol/l CaCl_2_ at 37 °C. Time to clot formation was recorded and values converted to arbitrary units (AU) by comparison with a human brain TF calibration curve covering clotting times from 20 to 600 s, corresponding to 1000 and 0 AU, respectively. Experiments were run in triplicate and averaged. As contaminating platelets may contribute to PCA [24], we performed the clotting assay in PBMC-free preparations in which PCA was undetectable (data not shown). Preliminary experiments had also confirmed the procoagulant effect of ATII (10^−6^M) added to PBMCs exposed to either NG (from 0.005 ± 0.002 to 0.01 ± 0.006 AU, *n* = 14, *p* < 0.01) or HG (from 0.011 ± 0.007 to 0.02 ± 0.01 AU, *n* = 10, *p* < 0.05).

#### TF antigen (Ag)

Cells were disrupted by three repeated freeze–thaw cycles and debris pelleted by centrifugation at 100xg for 1 h at 4 °C and supernatants used for ELISA (Imubind TF kit Sekisui Diagnostics, West Malling, United Kingdom). TF Ag levels were expressed in pg/ml using a reference curve created by the TF standards. Within and between assay variability was 3.5 and 5.5 %, respectively.

#### TF mRNA

Total RNA was extracted from PBMCs using the RNeasy mini kit (Qiagen, Hilden, Germany). RNA concentration and purity were determined by optical density measurement via Nanodrop (Thermo Fisher Scientific, Wilmington, Delaware USA). A mixture of 0.5 ng total RNA per sample was retro-transcribed with random primer-oligodT into complementary DNA (cDNA) using the Quantitect Reverse Transcription Kit (Qiagen, Hilden, Germany). The retro-transcription cycle was performed at 25 °C for 5 min, 42 °C for 30 min and 95 °C for 3 min. RealTime-PCR was carried out in a iQ5 Real Time PCR System and SsoAdvanced Sybr Green Supermix (Bio-Rad Laboratories, Hercules, CA) was employed on the basis of the manufacturer’s conditions: 95 °C, 30s; 40 cycles 95 °C, 5 s, 60 °C, 15 s;a final melting protocol with ramping from 65 °C to 95 °C with 0,5 °C increments of 5 s was performed. The primers sequence for RealTime-PCR were: TF, sense 5’-TTGGCAAGGACTTAATTTATACAC-3’, antisense 5’-CTGTTCGGGAGGGAATCAC-3’; GAPDH, sense: 5’-CCCTTCATTGACCTCAACTACATG-3’ and antisense: 5’-TGGGATTTCCATTGATGACAAGC-3’ (Invitrogen, Monza, Italy). All samples were analysed in duplicate and averaged. The relative expression of the target gene was normalized to the level of GAPDH in the same cDNA.

### Experimental design

#### Effect of HG

TF PCA, Ag and mRNA were assessed in resting and LPS-stimulated PBMCs exposed to either NG or HG. To evaluate the involvement of NF-kB in that setting, TF PCA was assessed in LPS-stimulated PBMCs exposed to either NG or HG and pretreated with BAY 11–7082 (10^−5^ M Sigma, Milan, Italy), a NF-kB inhibitor [25], or not.

#### Effect of AT2R agonism by C21 and AT1R blockade by OLM

Either C21, a selective AT2R agonist [19], or OLM, a selective AT1R antagonist [20], were added at log-increasing steps (10^−8^-10^−5^ M for both) to LPS-activated PBMCs incubated in either NG or HG media.

#### Effect of AT2R antagonism

To confirm its AT2R selectivity, C21 was added at log-increasing steps (10^−8^-10^−5^ M) to LPS-activated PBMCs incubated in NG or HG media in absence or presence of PD123,319 (10^−6^ M, di(trifluoroacetate) salt hydrate, Sigma Milan, Italy), a selective AT2R antagonist [26].

### Statistics

Statistical differences were assessed by non parametric tests (Wilkoxon’s and Mann-Whitney for paired and unpaired comparisons respectively) on either absolute data or percent changes from control conditions, these latter taken as a measure of drug effect. Data were reported as means ± SD unless otherwise reported. A two-tailed p-level <0.05 was the threshold for statistical significance.

## Results

### Effect of HG on TF expression

**Fig. 1:**
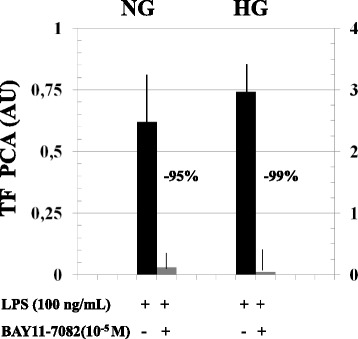
Abolition by BAY11-7082 (10^−5^M), a NF-kB inhibitor [23], of LPS-induced Tissue Factor procoagulant activity (TFPCA) in both normal (NG, 5.5 mM) and high (HG, 50 mM) glucose conditions. Note the different scale of the left and right ordinate. Means ± SD, *n* = 7 per group

Resting and LPS-stimulated TF PCA, Ag and mRNA were greater in HG than NG (Table 1). In both experimental conditions, BAY 11-7082 (10^−5^M) abolished LPS-stimulated PCA (Fig. 1).

**Table 1:** Resting and LPS(100 ng/ml)-stimulated Tissue Factor (TF) procoagulant activity (PCA), antigen(ag) and mRNA in normal (NG, 5.5 mM) and high (HG, 50.0 mM) glucose conditions

TF PCA (AU)		
	NG	HG
Resting	0.0054 ± 0.003	0.035 ± 0.4*
LPS-stimulated (*N* = 33)	0.93 ± 0.36**	2.7 ± 1.1*, **
TFAg (ng/mL)		
Resting	32 ± 24	428 ± 135*
LPS-stimulated (*N* = 12)	1486 ± 388**	2652 ± 622*, **
TF mRNA (normalized fold expression)		
Resting	0.007 ± 0.003	0.158 ± 0.1*
LPS-stimulated (*N* = 8)	0.65 ± 0.5**	0.85 ± 0.5*, **

*p < 0.001 HG vs NG, **p < 0.001 LPS- stimulated vs resting

### Effect of HG on C21 and OLM

**Fig. 2:**
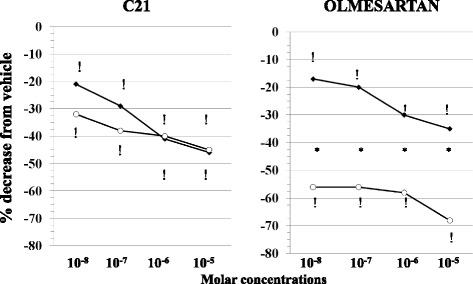
Concentration-dependent downregulation of LPS (100 ng/ml)-induced Tissue Factor procoagulant activity by C21 (*left panel*) and olmesartan (*right panel*) in normal (NG,♦, 5.5 mM) and high (HG,○, 50 mM) glucose conditions. *N* = 12 experiments each, ^!^ *p* < 0.001 vs vehicle, *p < 0.01 HG vs NG. For the sake of clarity, the graphs report only mean values

Both C21 and OLM downregulated LPS-induced TF PCA (Fig. 2). However, the extent of inhibition exerted by C21 was by and large not influenced by D-glucose concentrations (Fig. 2, left panel) in contrast with OLM whose effect was potentiated in HG (Fig. 2, right panel).

**Fig. 3:**
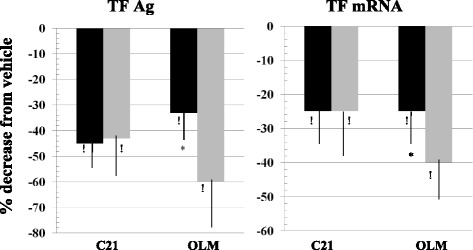
Downregulation of LPS(100 ng/ml)-induced TFAg (*Left panel*) and mRNA (*Right panel*) expression by C21 (10^−5^M) and olmesartan (OLM, 10^−5^M) in normal (NG, *black bars*, 5.5 mM) and high (HG, *gray bars*, 50 mM) glucose conditions. Means ± SD, at least *n* = 7 experiments per series, ! *p* < 0.001 vs. vehicle; *p < 0.001 HG vs NG

A similar behaviour was found when analyzing the effect of the two drugs on TFAg and TFmRNA (Fig. 3, left and right panel respectively).

### Effect of AT2R antagonism

**Fig. 4:**
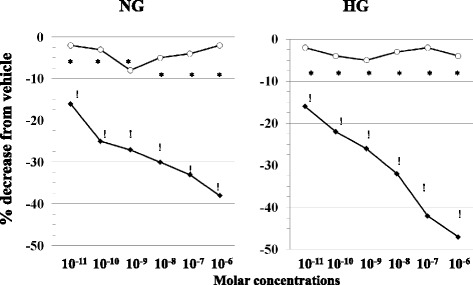
Antagonism by PD123,319 (○,10^−6^M), a selective AT2R blocker [26], of C21-mediated inhibition (♦) of LPS(100 ng/ml)-induced Tissue Factor procoagulant activity in normal (*left panel,* NG, 5.5 mM) and high (*right panel,* HG, 50 mM) glucose conditions. Mean ± SD, *n* = 6 experiments per group. ! *p* < 0.001 vs. vehicle; *p < 0.001 C21 (♦) vs C21+ PD123,319 (○)

PD123,319 (10^−6^M) abolished the effect of C21 in both NG and HG (Fig. 4, left and right panel respectively).

## Discussion

### Background of the study

The interpretation of the results of this study are facilitated by some further discussion of the involvement of ATII in innate immunity, a biological system by which germline-encoded receptor proteins recognize specific patterns presented by groups of pathogens [20]. Among them, LPS, the proinflammatory stimulus used in our experimental series, constitutes a major marker for the recognition of intruding Gram-negative bacteria that, by binding to Toll-like receptor(TLR)4 s and CD14 and recruiting adaptor proteins to the cytoplasmic Toll/interleukin-1 receptor, initiates the pathogen-induced inflammatory response of which activation of coagulation is a prominent component [1, 20]. ATII contributes to the innate immune response through complex and interrelated transcriptional and posttranscriptional mechanisms including upregulation of the TLR4 expression leading to a more intense NF-kB activation e.g. [27–29]. In turn, LPS or its intracellular proxies such as TNF-alpha [20] stimulate ATII generation [30] by activating renin, the earlier step of its biosynthetic chain [31], and increasing the number of ATII receptors available for stimulation by the peptide [32]. Thus, immunomodulation appears as an integral component of RAS functions far away from the conventional view of a hormonal system mainly involved in systemic blood pressure and body fluid volume control.

Although primarily aimed at the acute defence against infection and tissue damage, a growing body of evidence implicates innate immunity in several chronic conditions including type 2 diabetes [33] in which raised glucose concentrations, the hallmark of that disease, induce overexpression of Toll-like receptor(TLR) [34, 35] and CD14 [36], promote *de-novo* local synthesis of RAS components [37, 38] and potentiate a wide array of ATII-mediated biological actions e.g. [39–41]. By stimulating NADPH oxidase and mitochondrial metabolism, HG also accelerates reactive oxygen species (ROS) generation activating NFkB [16–18], thus initiating TF gene transcription along with a host of other proinflammatory cytokines. That concept is fully in line with our results showing upregulation by HG of both quiescent and LPS-induced TF PCA, mRNA and Ag expression and abolition of activated PCA by BAY11-7082, a NF-kB inhibitor [25], demonstrates the complete dependency upon the NFkB signalling pathway of the procoagulant effect of endotoxin in human PBMCs.

### HG and AT2R agonism on LPS-stimulated TF expression

Within the frame of reference summarized in the previous paragraph, our data confirm the inhibitory action of AT2R agonism by C21 on LPS-stimulated TF expression in human PBMCs [14] and the complete antagonism exerted by PD123,319 reassures about the specificity of the response. The parallel decrease of TFAg and mRNA is consonant with transcriptional modulation of TF protein likely by NFkB downregulation [41], an interpretation fully supported by recent reports showing attenuated cytokine production by C21 in LPS-stimulated monocytic cells [42]. In addition to those relevant albeit confirmatory findings, however, the main and original pathophysiological implications of this study arise from the contrast between the neutral effect of HG on C21-induced TF inhibition and the potentiated effect of AT1R blockade by OLM in PBMCs exposed to high D-glucose. That divergent behaviour, in fact, indicates that HG impacts differently on AT1R- and AT2R-mediated signal transduction pathways although our data do not allow any obvious explanation for that divergence. On a speculative basis, increased AT1Rs available for binding as a consequence of higher D-glucose concentrations [32] might potentiate the effect of OLM although the same should theoretically apply to AT2R stimulation given the sensitivity of that receptor subset to glucose concentrations [43]. The greater inhibitory effect of OLM might also relate to increased ANGII production in PBMCs grown in HG media [37, 38] although AT2R antagonism by PD 123, 329 per se was totally neutral on the procoagulant potential of PBMCs in our previous experience [14]. Thus, TF inhibition by C21 may merely represent the result of a pharmacological manipulation of physiologically silent binding sites activated by an agonist attaining concentrations at the receptor site far exceeding those achieved by ATII, the endogenous ligand [44]. Other mechanisms peculiar to AT1R blockers may also be at play including modulation of TLR4 expression and activity possibly independent of AT1R blockade [45] but this as well as all the above outlined possibilities are speculative and our data cannot provide any solid evidence in favour or against them.

## Conclusion

This study confirms the stimulating property of HG on resting and activated procoagulant activity and demonstrate the obligatory role of NFkB in mediating the procoagulant effect of LPS in human PBMNCs. In addition, we showed the neutral effect of HG on the TF-inhibiting effect of C21, a selective AT2R agonist, quite in contrast with the potentiated effect of OLM under the same experimental conditions, a divergent behaviour indicating that HG impacts differently on AT1R- and AT2R-mediated signal transduction pathways. However, further work is needed to understand the precise determinants of the phenomenon more precisely and the extent to which in-vitro data can be transferred to in-vivo conditions.
